# Synchronous papillary renal neoplasm with reverse polarity and multilocular cystic renal neoplasm of low malignant potential in unilateral kidney: case report with molecular analysis and literature review

**DOI:** 10.3389/fonc.2025.1605192

**Published:** 2025-08-11

**Authors:** Pichayut Nithagon, Joeffrey Chahine, Lambros Stamatakis, Rashmi Samdani

**Affiliations:** ^1^ Department of Pathology and Laboratory Medicine, Medstar Georgetown University Hospital, Washington, DC, United States; ^2^ Medstar Health Urology Oncology, Medstar Georgetown University Hospital, Washington, DC, United States

**Keywords:** papillary renal neoplasm with reverse polarity, multiloculate cystic renal neoplasm of low malignancy potential, synchronous renal tumors, KRAS mutation, renal neoplasm pathogenesis

## Abstract

Papillary Renal Neoplasm with Reverse Polarity (PRNRP) is a rare renal tumor, recently described in 2019 by Al-Obaidy et al. defined by characteristic histology of papillary neoplasm with apically located WHO/ISUP grade 1nuclei and frequent *KRAS* mutations. Multilocular cystic renal neoplasm of low malignant potential (MC-LMP) is an indolent tumor with a characteristic multicystic appearance with cysts lined by WHO/ISUP nuclear grade1 clear cells and presence of *VHL* alterations similar to that of clear cell renal cell carcinoma (ccRCC); therefore, considered its variant. Simultaneous occurrence of both these tumor types that are immunophenotypically and genetically distinct within same kidney is extremely rare and this is the first case report to date. Herein, we report a case of a 70-year-old male who was incidentally found to have bilateral renal cysts on imaging follow up for cardiovascular problems. The diagnosis of PRNRP and MC-LMP within the same kidney was made on histology in conjunction with ancillary tests. Awareness of PRNRP and MC-LMP is crucial for accurate diagnosis, as these tumors often resemble some of the aggressive variants of Renal cell carcinoma (RCC), such as Papillary RCC (pRCC) and ccRCC respectively on histology. Ability to correctly identify these indolent tumors is essential for optimal treatment options as they are often amenable to partial nephrectomy. This case underscores the need for further research into the pathogenesis and clinical implications of synchronous renal tumors with distinct immunophenotypes, and genomic profiles within the same kidney.

## Introduction

Renal cell carcinoma (RCC) accounts for 4.1% of all new cancers and 2.4% of all cancer-related deaths according to SEER (Surveillance, Epidemiology, and End Results Program) data as of the most recent report (1). RCC has been historically classified based on histomorphologic and cytomorphologic features as tumors with light/clear cell staining cytoplasm, tumors showing papillary or tubulopapillary architecture, tumors with granular/-eosinophilic cytoplasm, tumors with spindle cell morphology, poorly differentiated carcinoma, and tumors featuring distinct genotypic and immunophenotypic profiles. Papillary Renal Neoplasm with Reverse Polarity (PRNRP) is a recently described renal tumor based on its unique histology and molecular profile. Despite its indolent behavior, the World Health Organization (WHO) 2019 RCC classification has not recognized it as a distinct entity and has placed it under the category of Papillary RCC (pRCC), which is a far more aggressive tumor with a worse prognosis. The key defining histologic features include papillary or tubular architecture, WHO/ISUP grade 1 nuclei positioned toward the apex with an oncocytic appearance, a unique immunophenotypic profile with strong diffuse GATA3 expression, and negative Vimentin staining (2–4). Molecularly, these tumors have *KRAS* mutation in codon 12, distinguishing them from pRCCs, which do not carry these mutations and instead show karyotypic abnormalities characterized by trisomy of chromosomes 7, 17, and loss of chromosome Y (3). Conversely, multilocular cystic renal neoplasm of low malignant potential (MC-LMP) is a WHO-recognized type of renal cell tumor showing a multicystic appearance with cysts lined by WHO/ISUP nuclear grade 1 clear cells. These tumors carry an indolent clinical course. The simultaneous occurrence of renal tumors with distinct histologic types and genetics is rare, though there are a few case reports of such instances. Since recognition of PRNRP, a subset is known to occur synchronously with other renal tumors. However, a case of synchronous PRNRP with MC-LMP has yet to be reported.

To our knowledge, this is the first documented case of incidental synchronous occurrence of PRNRP and MC-LMP within the same kidney that requires combination of morphologic characteristics, immunohistochemistry and molecular analysis to establish diagnosis. This case report further contributes to the diverse presentation of PRNRP and offers insight into molecular findings, prognosis and clinical outcomes of these neoplasms with a review of the literature.

## Case description

A 70-year-old male with a notable history of hypertension, hyperlipidemia, and aortic stenosis—who had previously undergone a prosthetic aortic valve replacement—was found to have bilateral renal cysts incidentally during follow-up imaging for his aortic valve replacement. The patient was asymptomatic with the lesions but was further assessed with an abdominal MRI.

**Figure 1:**
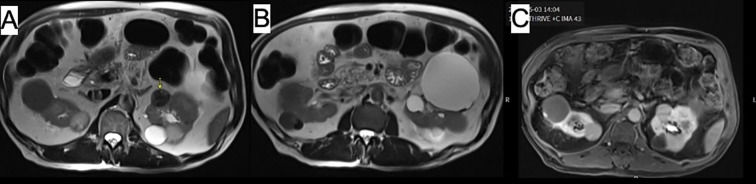
Abdominal MRI images showing **(A, B)** mass within anterior upper pole of left kidney with thick irregular internal septation (arrow). Image **(C)** demonstrates additional cysts in both kidneys.

The imaging showed a 2.5 cm complex renal mass within the upper to mid pole of the left kidney with irregular, thickened septal enhancement on contrast images, which likely represents a Bosniak type III lesion. There were additional Bosniak type II/IIF lesions within both kidneys. There was no evidence of abdominal adenopathy (**Figure 1**).

Given the clinical presentation of bilateral renal cystic lesions concerning for primary renal neoplasm, a staged surgical approach was recommended. Intraoperative renal ultrasound was performed, demonstrating a 3 cm cystic anterior renal mass just superior to the renal hilum which corresponded to the position seen on the MRI. Due to the larger solid component in the anterior cystic mass, a robotic partial nephrectomy was advised. In addition, within the same kidney was an anterior middle-pole complex renal cyst. Decortication of that large anterior mid-pole renal cyst was performed to enhance visualization and facilitate the dissection of the cystic lesion, followed by a partial nephrectomy for the mass.

## Histology

**Figure 2:**
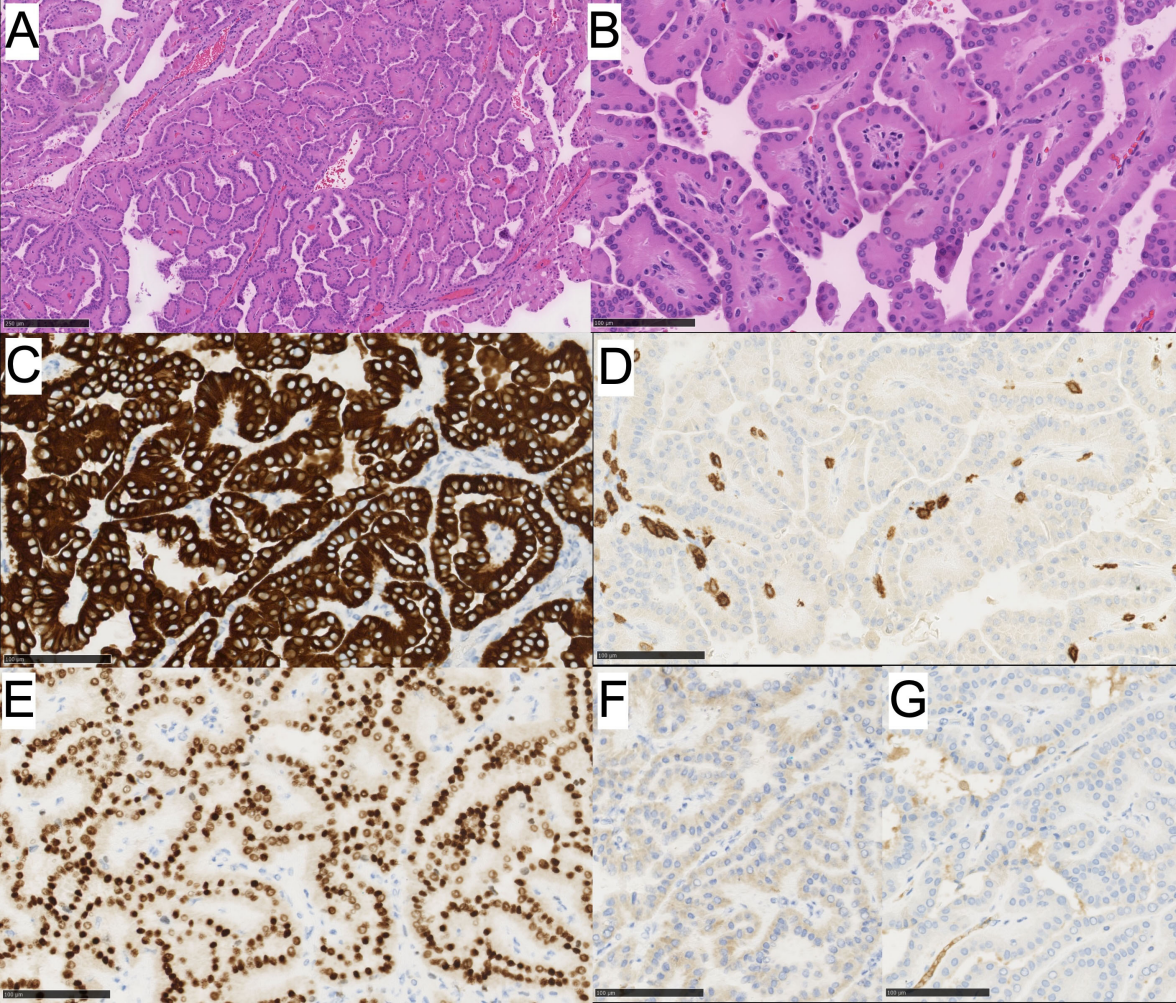
**(A)** PRNRP showing oncocytic cells with low-grade reversed nuclei polarity and papillary architecture and **(B)** higher power image. Immunohistochemistry for **(C)** CK7 is positive, **(D)** CD117 is negative, **(E)** GATA3 shows positive nuclear staining, **(F)** CAIX is negative, and **(G)** AMCAR is negative.

Gross examination of the left renal partial nephrectomy revealed a well-circumscribed and encapsulated mass measuring 1.7 x 1.7 x 1.0 cm. Upon sectioning, the cut surface appeared tan, brown to tan-pink with a loose papillary architecture. There was no evident necrosis or gross invasion of the capsule. On histological examination, the tumor was solid and cystic, exhibited a papillary architecture, and had WHO ISUP grade 1 nuclei with oncocytic cytoplasm and apically located nuclei (**Figures 2A, B**). Based on the morphologic characteristics, our differential was broad, ranging from benign to malignant categories. These included pRCC (malignant neoplasm), oncocytoma (benign neoplasm), PRNRP, and eosinophilic variant of ccRCC (malignant neoplasm).

To differentiate these tumors, an immunohistochemical work-up was performed for the following markers: CK7, CK20, GATA3, PAX8, CA-IX, and CD117 (**Table 1**). The neoplastic cells were diffusely and strongly positive for CK7, GATA3, PAX8 (weak positivity), negative for racemase, CA-IX, CD117, and CK20 (**Figures 2C-G**). The immunoprofile in conjunction with morphology best classifies this neoplasm as a Papillary Renal Neoplasm with reverse polarity.

**Table 1:** Immunohistochemical profiles of Renal tumors in the differential diagnosis of PRNRP.

Immunohistochemistry	PRNRP	Papillary RCC	Oncocytoma	Clear cell RCC eosinophilic variant
CK7	+	+/-	–	–
GATA3	+	–	–	–
PAX-8	+	+	+	+
Racemase/CA-IX	–	-/focally +	–	+
CD117	–	–	+	–

To confirm our findings, a targeted *KRAS* mutation analysis using real-time PCR (RT-PCR ROCHE LSR v2) was performed. *KRAS* codon G12C (c.34G>T) mutation in exon 2 was detected.

**Figure 3:**
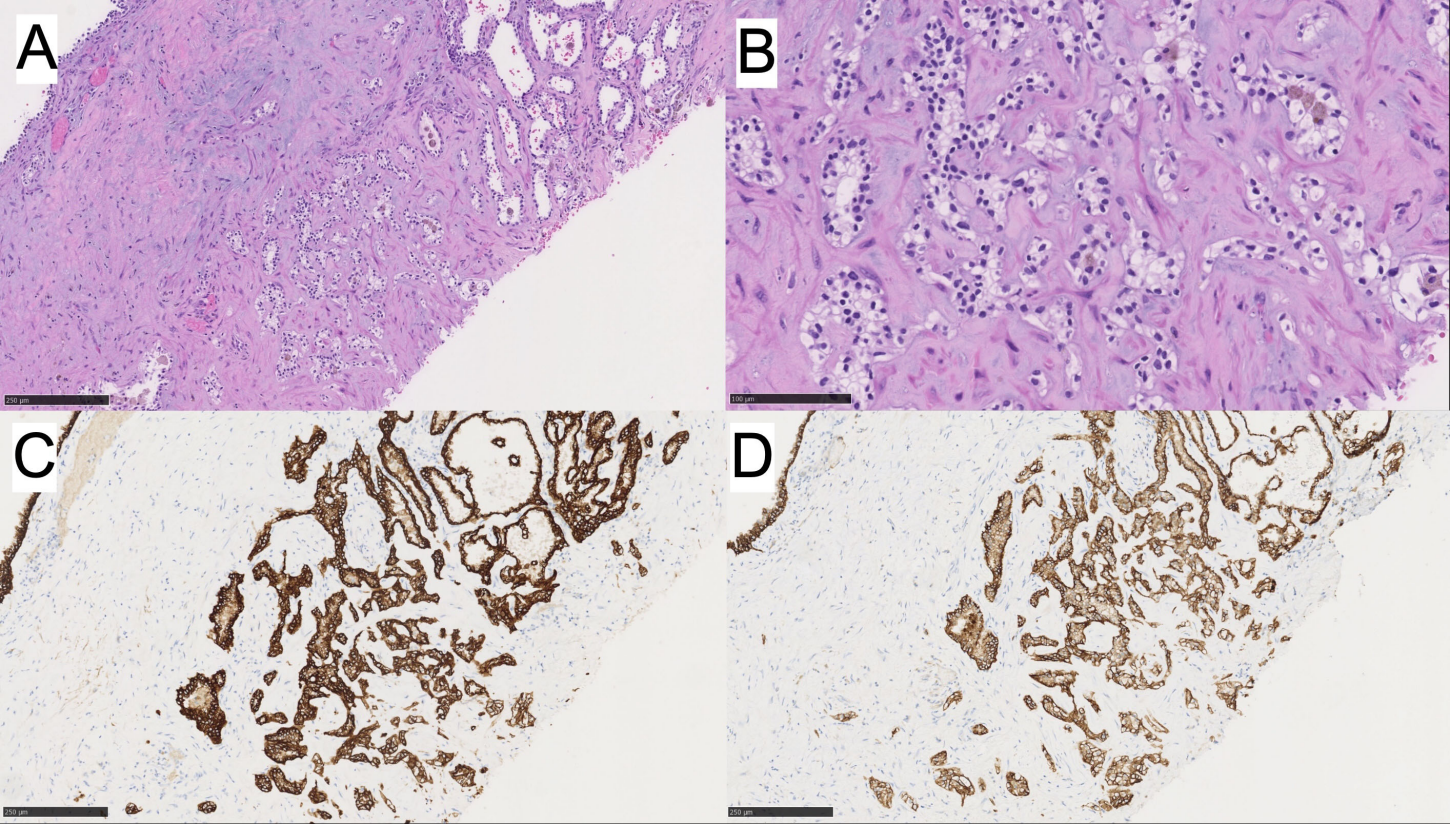
**(A, B)** MC-LMP with higher power. Immunohistochemistry for **(C)** CK7 is positive, and **(D)** CAIX is positive.

In addition, within the same kidney was an anterior middle-pole complex renal cyst that underwent decortication intraoperatively, and the cyst wall fragments were submitted for pathology evaluation. On histology, these fragments had multiple thin, fibrous septa lined by clear cells with few foci of clear cell clusters. The nuclei were uniformly low grade (WHO/ISUP grade 1) with no nodular expansion (**Figures 3A, B**). Immunophenotypically, these neoplastic clear cells were positive for CK7 and CA-IX (**Figures 3C, D**). The morphology and immunophenotype of the cyst were consistent with MC-LMP.

Overall, our findings feature two morphologically and immunophenotypically distinct tumor types; PRNRP and MC-LMP within the same kidney.

## Molecular analysis

To further confirm the histologic impression and identify a plausible association of simultaneous occurrence of these two tumors in the same kidney, after appropriate patient consent, Next generation sequencing (NGS) and karyotype analysis were performed. A pathogenic variant in *KRAS* (Exon 2, p.G12C, c.34G>T) was detected with a variant allele frequency (VAF) of 30% in the PRNRP. No mutations in the *KRAS* gene were found in MC-LMP; instead, the tumor showed a frameshift variant in the *BCR* gene (c.3275_3278dupCCGG, p.V1094fs) with a VAF of 16%. The *BCR* gene is one of the candidate genes used to differentiate MC-LMP from ccRCC with cystic change.

No other genetic alterations associated with syndromic or synchronous renal tumors (i.e. *VHL, MET, FH, SDH, TSC1/TSC2, BAP1*) were identified in the MC-LMP tumor sample. The morphology, immunophenotype, PCR, and NGS findings, indicate that these two tumors are genetically distinct and incidental synchronous tumors with no syndromic association. Based on the clinical follow-up thus far, it appears to carry an indolent prognosis.

On follow up, the patient has recovered well from surgery and is doing well post-surgery. His follow up recent CT chest abdomen pelvis 9 months post resection did not demonstrate any metastatic disease or residual tumor within bilateral renal cysts.

## Discussion

The frequency of multifocality in renal tumors is about 5.3% (5). Multifocal renal tumors can be histologically and genetically concordant or discordant and are common in day-to-day practice as well as reported in the literature with a combination of tumor types including pRCC, ccRCC, Chromophobe RCC, MC-LMP, Oncocytoma, etc. The rate of discordant tumor types in multifocal renal tumors is about 6-30%. Their pathogenesis is often multifactorial and yet to be fully elucidated (5–8).

Synchronous and multifocal renal tumors are often seen in association with genetic syndromes like von Hippel-Lindau (VHL) disease or hereditary papillary RCC. About 5-13% of sporadic MC-LMP are found to have concurrent ccRCC, with the likely etiology being the *VHL* gene mutations. Previously, a subset of PRNRP cases had been noted to occur synchronously with an additional renal tumor in the ipsilateral kidney, including pRCC, ccRCC, chromophobe RCC, and oncocytoma. To our best knowledge, there is no previously reported association between PRNRP and MC-LMP (2). Synchronous PRNPR were typically independent tumors, often physically separate nodules. This reflects the possibility that PRNRP can arise as a sporadic collision tumor alongside others. In a case report with molecular analysis by Lee et al. showed a case of PRNRP harboring a KRAS p.G12V mutation while the synchronous ccRCC carried a distinct *PIK3CA* mutation (9), suggesting two clonally independent tumors in one kidney. Additionally, a case of PRNRP with KRAS mutation was also reported synchronously with urothelial carcinoma with a distinct *FGFR3/KDM6A* mutation (10). Similarly, our case demonstrated non-overlapping mutation; *KRAS* mutation in PRNRP and *BCR* mutation in MC-LMP, further supporting the concept of genetically separate lesions occurring synchronously.

A striking immunohistochemical feature of PRNRP is it’s consistent GATA3 expression. GATA3 immunohistochemical stain although non-specific can be expressed in a variety of tumor types; in the appropriate clinical setting, it is helpful for confirming urothelial or mammary origins. GATA3 is a transcription factor involved in the development of the urinary tract (11). In a normal kidney, GATA3 is expressed in certain kidney structures (distal nephron tubules and collecting ducts) but not in proximal tubules (12). Although GATA3 expression in kidney tumors is not well documented, there appears to be a pattern of GATA3 expression in tumors believed to originate from the distal tubules and collecting ducts. Among renal tumors, the highest expression is observed in PRNRP and low-grade oncocytic tumors (LOTs), both of which shows 100% GATA3 positivity. Fumarate hydratase-deficient RCC shows GATA3 expression in 33% of cases, though staining may be focal. Clear-cell papillary renal tumors (CCPRT) exhibit 41-67% GATA3 positivity, a characteristic feature, in addition to the “cup-like” CAIX staining pattern, that differentiates them from ccRCC. Collecting duct carcinoma (CDC) shows positivity in 33% of case. Chromophobe RCC expresses GATA3 in 6-51% of cases, while 9% of TF3-translocated RCC shows GATA3 positivity (13). In contrast, ccRCC and pRCC both thought to arise from proximal nephrons are negative for GATA3 expression (14). The consistent expression of GATA3 in PRNRN possibly suggest its origin in distal tubules. While GATA3 can aid in distinguishing specific renal tumor subtypes, its use should be limited to a panel of markers rather than as a sole diagnostic tool.

*KRAS* mutations are considered a central part of the pathogenesis of adenocarcinomas of many organs including lungs, colorectal, and pancreas (15). *KRAS* mutations are also frequently reported in various papillary or mucinous precursor lesions such as intraductal papillary mucinous neoplasms of the pancreas or urothelial papilloma/carcinomas. *KRAS* mutations have also been implicated in approximately 5% of urothelial carcinoma but is not considered a central part of its pathogenesis (16). Upper urothelial tract carcinoma and lower urothelial tract carcinomas have demonstrated mutational differences. *RAS* associated alterations are seen more commonly in the upper urothelial tract carcinomas (17). *KRAS* mutations are rare in clear-cell renal cell tumors, except for one case reported in the literature thus far (8). The overall frequency of *KRAS* mutation in PRNRP is about 85% (2, 18, 19). and the most common mutation being *KRAS* p.G12V (54%) (18). In contrast our case showed p.G12C (c.34G>T) *KRAS* mutation.

*BCR* mutation is a key part of the pathogenesis of chronic myeloid leukemia, the *BCR-ABL1* fusion (Philadelphia chromosome), where the upregulation of tyrosine kinase activity drives leukemic cell proliferation. *BCR* mutations are infrequent in solid tumors; however, its alterations have been linked to certain renal tumors, particularly MC-LMP. A frameshift alteration detected in the *BCR* gene in MC-LMP and no *VHL* gene mutation compared to ccRCC suggests likely separate clonal evolutionary mechanisms (19), despite the two neoplasms sharing overlapping histomorphology. MC-LMP often lack the typical VHL alterations (only approximately 25% show VHL alterations) that are seen in ccRCC (20). In fact, MC-LMP often shows other genetic changes in genes such as TCEB1 or other non-VHL-associated. The absence of VHL alterations supports the classification of MC-LMP as a distinct entity from ccRCC and further confirming its indolent nature.

In our case, the absence of end-stage renal disease (ESRD) is notable, as many renal tumors, particularly cystic neoplasms, are frequently identified in patients with ESRD due to alterations in the renal microenvironment (21–23). Acquired cystic kidney disease (ACKD) develops in a significant proportion of ESRD patients – ranging from 8-95% in some studies (24, 25) in dialysis patients – and increases the risk of cystic renal neoplasms. Incidence of renal cancers is approximately 50 times greater in ACKD patients than in the general population (26, 27). Cystic neoplasms observed in ESRD include acquired cystic disease-associated renal cell carcinoma (ACD-RCC), clear cell papillary renal tumor and pRCC (23, 28–30). In contrast, ESRD patients without ACKD are less prone to developing cystic neoplasms. The lack of ESRD in our patient suggests that the coexistence of these two tumors is unlikely driven by the same environmental or systemic factors that contribute to cystic neoplasms in ESRD patients (31).

Awareness of PRNRP and MC-LMP is critical for accurate diagnosis and optimal treatment. PRNRP can be misdiagnosed with other papillary tumors, most importantly pRCC which carries a far worse prognosis than PRNRP. Therefore, awareness of this entity is important to prevent overtreatment. Given its indolent nature, nephron-sparing approaches may be appropriate when feasible. Similarly, MC-LMP should not be confused with more aggressive renal neoplasms such as ccRCC with cystic change as often they are found in association with them. MC-LMP carries an excellent prognosis when completely excised (32–36).

Treatment strategies for both tumors are largely surgical, with no role for adjuvant therapy in the absence of adverse features or metastatic disease. However, the rare coexistence of these tumors emphasizes the importance of thorough histologic evaluation and molecular profiling, which can guide management decisions and improve outcomes. Our case demonstrates absence of recurrence or metastasis during the 9-months follow-up period, which align with the reported indolent nature of these neoplasms (**Table 2**).

**Table 2:** Literature review of synchronous PRNRP and other renal neoplasms with treatment approaches and follow-up.

Case	Synchronous tumors	Treatment	Outcome	Reference, year, PMID
1	PRNRP + ccRCC	Radical nephrectomy	NED (8 months follow-up)	Lee et al. (2020) (9), PMID 33023600
2	PRNRP + urothelial carcinoma	Nephroureterectomy and chemotherapy	NED (3 months follow-up)	Li et al. (2023) (10), PMID 37924117
3	PRNRP + MC-LMP	Partial nephrectomy	NED (9 months follow-up)	Current case
4	PRNRP + pRCC	Total nephrectomy	NED (20 months follow-up)	Al-Obaidy et al. (2022) (2), PMID 35152262
5	PRNRP + pRCC	Total nephrectomy	NED (160 months follow-up)	Al-Obaidy et al. (2022) (2), PMID 35152262
6	PRNRP + pRCC	Total nephrectomy	DOC (113 months follow-up)	Al-Obaidy et al. (2022) (2), PMID 35152262
7	PRNRP + ccRCC	Partial nephrectomy	NED (134 months follow-up)	Al-Obaidy et al. (2022) (2), PMID 35152262
8	PRNRP + oncocytoma	Total nephrectomy	NED (114 months follow-up)	Al-Obaidy et al. (2022) (2), PMID 35152262
9	PRNRP + ccRCC	Total nephrectomy	NED (105 months follow-up)	Al-Obaidy et al. (2022) (2), PMID 35152262
10	PRNRP + ACD-RCC	Total nephrectomy	NED (7 months follow-up)	Al-Obaidy et al. (2022) (2), PMID 35152262
11	PRNRP + multiple oncocytoma	Total nephrectomy	NED (94 months follow-up)	Al-Obaidy et al. (2022) (2), PMID 35152262
12	PRNRP + oncocytoma	Total nephrectomy	NED (87 months follow-up)	Al-Obaidy et al. (2022) (2), PMID 35152262
13	PRNRP + pRCC	Total nephrectomy	NED (87 months follow-up)	Al-Obaidy et al. (2022) (2), PMID 35152262
14	PRNRP + ccRCC + ccPRCC	Partial nephrectomy	DOC (5 months follow-up)	Al-Obaidy et al. (2022) (2), PMID 35152262
15	PRNRP + chromophobe RCC	Partial nephrectomy	NED (45 months follow-up)	Al-Obaidy et al. (2022) (2), PMID 35152262
16	PRNRP + ccRCC	Partial nephrectomy	NED (30 months follow-up)	Al-Obaidy et al. (2022) (2), PMID 35152262
17	PRNRP + oncocytoma	Partial nephrectomy	NED (7 months follow-up)	Al-Obaidy et al. (2022) (2), PMID 35152262
18	PRNRP + ACD-RCC	Total nephrectomy	NED (10 months follow-up)	Al-Obaidy et al. (2022) (2), PMID 35152262
19	PRNRP + ACD-RCC	Total nephrectomy	NED (16 months follow-up)	Al-Obaidy et al. (2022) (2), PMID 35152262
20	PRNRP + pRCC	Total nephrectomy	DOC (3 months follow-up)	Al-Obaidy et al. (2022) (2), PMID 35152262
21	PRNRP + pRCC	Partial nephrectomy	NED (15 months follow-up)	Al-Obaidy et al. (2022) (2), PMID 35152262

Our case underscores the need for further studies to elucidate the potential biological links between these rare renal neoplasms and refine diagnostic and therapeutic approaches.

## Data Availability

The original contributions presented in the study are included in the article/supplementary material. Further inquiries can be directed to the corresponding author.
